# Corrigendum to: ‘Curvature-induced stiffening of a fish fin’ (2022) by Nguyen *et al.*

**DOI:** 10.1098/rsif.2022.0880

**Published:** 2023-01-11

**Authors:** Khoi Nguyen, Ning Yu, Mahesh M. Bandi, Madhusudhan Venkadesan, Shreyas Mandre

*J. R. Soc. Interface* **14**, 20170247. (Published online 31 May 2022) (https://doi.org/10.1098/rsif.2017.0247)

This corrigendum corrects the record and includes Mahesh M. Bandi and Madhusudhan Venkadesan as corresponding authors for the article.

bandi@oist.jp

m.venkadesan@yale.edu

shreyas.mandre@warwick.ac.uk

